# Seasonal climatic niche and migration movements of Double‐crested Cormorants

**DOI:** 10.1002/ece3.9153

**Published:** 2022-08-23

**Authors:** D. Tommy King, Guiming Wang, Fred L. Cunningham

**Affiliations:** ^1^ U. S. Department of Agriculture, Wildlife Services National Wildlife Research Center Mississippi State Mississippi USA; ^2^ Department of Wildlife, Fisheries and Aquaculture Mississippi State University Mississippi State Mississippi USA

**Keywords:** climatic niche switching hypothesis, migration distance, migration speed, *Nannopterum auritum*, satellite tracking

## Abstract

Avian migrants are challenged by seasonal adverse climatic conditions and energetic costs of long‐distance flying. Migratory birds may track or switch seasonal climatic niche between the breeding and non‐breeding grounds. Satellite tracking enables avian ecologists to investigate seasonal climatic niche and circannual movement patterns of migratory birds. The Double‐crested Cormorant (*Nannopterum auritum*, hereafter cormorant) wintering in the Gulf of Mexico (GOM) migrates to the Northern Great Plains and Great Lakes and is of economic importance because of its impacts on aquaculture. We tested the climatic niche switching hypothesis that cormorants would switch climatic niche between summer and winter because of substantial differences in climate between the non‐breeding grounds in the subtropical region and breeding grounds in the northern temperate region. The ordination analysis of climatic niche overlap indicated that cormorants had separate seasonal climatic niche consisting of seasonal mean monthly minimum and maximum temperature, seasonal mean monthly precipitation, and seasonal mean wind speed. Despite non‐overlapping summer and winter climatic niches, cormorants appeared to be subjected to similar wind speed between winter and summer habitats and were consistent with similar hourly flying speed between winter and summer. Therefore, substantial differences in temperature and precipitation may lead to the climatic niche switching of fish‐eating cormorants, a dietary specialist, between the breeding and non‐breeding grounds.

## Graphical abstract

We investigated seasonal climate niches of migratory double‐creased cormorants. We used the satellite‐telemetry locations of the same birds between summer and winter. Our findings suggest that double‐crested cormorants switched climate niches between the breeding and non‐breeding grounds.

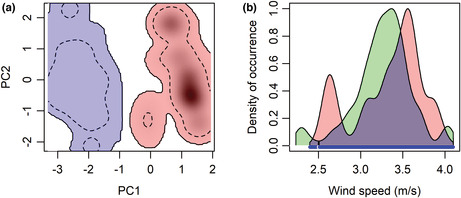

## 1 INTRODUCTION

Migration is the repeated seasonal movement of animals between the breeding and non‐breeding grounds (Newton, 2008). Climate, intra‐ and inter‐specific competition, and predation are believed to be the ecological drivers of the evolution of migration (Salewski & Bruderer, 2007). The fecundity selection hypothesis posits that migrants depart from their non‐breeding grounds for improving breeding opportunities during spring (Gordo, 2007). For instance, migratory birds can gain greater reproductive success in the high latitudes of longer daytime and higher productivity during summer than in their low‐latitude non‐breeding grounds (Salewski & Bruderer, 2007). On the contrary, winter climate on the breeding grounds in the northern temperate region is often too inclement for migratory birds to survive (Gómez et al., 2016). Migrants depart from their breeding grounds and migrate to the non‐breeding grounds for winter. The viability selection hypothesis predicts that autumn migration may enhance winter survival of migrants (Gordo, 2007; Somveille et al., 2015). Climatic conditions are dramatically different between the temperate breeding and tropical or subtropical non‐breeding grounds. Therefore, investigations of variation in seasonal climatic niche can provide essential information for understanding the ecology and evolution of migration.

Migratory birds are subjected to different climatic regimes along their migration routes and between breeding and non‐breeding grounds (Gómez et al., 2016; Marini et al., 2013; Winger et al., 2019). The dramatic differences in climatic conditions during the annual cycles represent physiological challenges to migrants. Avian migrants may be forced to depart from breeding grounds to avoid inclement winter climate in the northern temperate region (Gómez et al., 2016; Somveille et al., 2015). Avian migrants may track similar climatic conditions (i.e., climate tracking) between breeding and non‐breeding grounds (Gómez et al., 2016; Joseph & Stockwell, 2000; Nakazawa et al., 2004; Tingley et al., 2009), or migrants may adapt or switch to different seasonal climatic conditions (i.e., climate switching) (Gómez et al., 2016; Laube et al., 2015). Temporal variation and flexibility (e.g., niche shift) of ecological niche may allow organisms to adapt to environmental changes (Tingley et al., 2009; Winger et al., 2019). Previous studies have found evidence for both climatic tracking and switching strategies in migratory birds. About 73% of 49 migratory Parulidae species were climatic niche trackers (Gómez et al., 2016). On the contrary, 80% of 355 migratory birds migrating through Eurasian–African flyways were climatic niche switchers (Ponti et al., 2020).

Avian flight is energetically expensive, particularly over long distances (Blem, 2000). Birds can fly with favorable or optimal wind conditions such as tailwinds or thermal updrafts to gain additional energy for their movements (Chapman et al., 2011; Harel, Duriez, et al., 2016; Safi et al., 2013). Wind conditions affect the patterns of movements, migration decisions, and survival of birds (Huang et al., 2017; Safi et al., 2013; Thorup et al., 2006; Weimerskirch et al., 2012). Potentially migratory birds can use the same optimal flight strategies to adapt to wind conditions on both breeding and non‐breeding grounds. We hypothesized that migratory birds would select similar wind conditions (e.g., speed) on both breeding and non‐breeding grounds to reduce the energetic costs of flight. However, most studies have used species presence or museum specimen records to investigate seasonal climatic niches of migratory birds. Data on species presence lack information on the climatic niche and wind conditions of the same migrant populations between the breeding and non‐breeding grounds. Global positioning system (GPS) or satellite tracking data not only have high accuracy of locations, but also can be used to delineate the seasonal ranges of migrant populations.

The Double‐crested Cormorant (*Nannopterum auritum*, hereafter cormorant) is a piscivorous bird living in open water bodies such as lakes and large rivers, swamps, and wetlands (Aderman & Hill, 1995; Scherr et al., 2010). During the past three decades, cormorants have increasingly used freshwater commercial aquaculture facilities in the Southeastern United States (US), causing aquaculture losses to the commercial channel catfish (*Ictalurus punctatus*) operation (Burr et al., 2020; King et al., 2010; Scherr et al., 2010). Cormorants wintering in the Gulf of Mexico (GOM) migrate to the Northern Great Plains and Great Lakes areas for summers (King et al., 2012; Scherr et al., 2010). King et al. (2012) determined spring departure dates and arrival dates as the last day on the non‐breeding ground and the first day on the breeding ground, respectively, and recognized that the estimates of migration dates were coarse without delineating the boundaries of the breeding and non‐breeding grounds for each tracked individual. Scherr et al. (2010) used the areas occupied by cormorants from December to March as wintering areas and determined the departure and arrival dates of spring and autumn migration between the wintering areas and summer roost locations. Scherr et al. (2010) also investigated the autumn migration staging areas of cormorants. However, no studies have investigated seasonal variation in climate niche of cormorants.

In this study, we reanalyzed the satellite tracking data of the cormorants on the breeding and non‐breeding grounds from King et al. (2012) to estimate the seasonal niche dynamics of cormorants. The breeding grounds in the Northern Great Plains and Great Lakes areas have a colder autumn and winter climate than the non‐breeding grounds in the Southeastern US. Shallow water bodies at the breeding grounds are often frozen during winter, which substantially reduces winter food availability for fish‐eating birds if the birds remained on the breeding grounds over the winter. It is plausible to hypothesize that cormorants would switch climatic niches between the breeding and non‐breeding grounds as a behavioral adaption to the inclement climatic conditions on the northern template breeding grounds during winter. We tested the hypothesis that cormorants would be a climatic niche switcher because of substantial difference in climatic conditions between the non‐breeding grounds in the subtropical region and the breeding grounds in the northern temperate region. We also predicted that the wind conditions used by the cormorants would overlap between the breeding and non‐breeding grounds. Studies of seasonal climatic niche overlap or switch help researchers understand the adaptation and ecological drivers of the seasonal migration of cormorants.

## 2 METHODS

### 2.1 Cormorant captures and satellite transmitter attachment

This study reanalyzed satellite tracking data on the breeding and non‐breeding grounds to estimate the seasonal niche dynamics of cormorants (King et al., 2012). Cormorants were captured at night roost sites near aquaculture facilities in Alabama, Arkansas, Louisiana, and Mississippi (figure 1 of King et al., 2012) using a modified night‐lighting technique (King et al., 1994). Fifty‐three cormorants were fitted with 30‐ or 45‐g (about 0.2%–0.3% of cormorant body mass) satellite platform transmitter terminals (PTT; Microwave Telemetry, Inc.) using a modified backpack harness (King et al., 2012; King & Tobin, 2000). Satellite transmitters were programmed to record locations for six consecutive hours every 48 h from October through mid‐June and six consecutive hours every 10 days from mid‐June through September (King et al., 2012). Among the 53 tracked cormorants captured in 2000–2001, 22 birds produced enough data of at least one migration trip for model fitting and movement analysis (King et al., 2012). We reanalyzed the location data of 22 satellite‐tracked cormorants. All experimental protocols of animal capture and handling were approved by the United States Department of Agriculture (USDA), National Wildlife Research Center, and Institutional Animal Care and Use Committee (IACUC Protocol QA‐742).

### 2.2 Satellite relocation data processing

Satellite transmitters used in this study are known to have substantial location error (Vincent et al., 2002). We used a continuous‐time correlative random walk (CRW) model implemented with extended Kalman filter in the R package *crawl* to fit the entire movement trajectory of each tracked bird to reduce or filter location error (Johnson et al., 2008). Continuous‐time CRW models can be fit to location data with irregular time intervals. The CRW model *crwMLE* allows for different variances of location error for different Argos location classes (i.e., LC0, LC1, LC2, and LC3) (Johnson et al., 2008). We predicted the geographic coordinates of cormorant locations at the original relocation times to reduce relocation error. All filtered locations were used for determining spring departure day from non‐breeding grounds, spring arrival at breeding grounds, autumn departure day from breeding grounds, and autumn arrival at non‐breeding grounds. Since our data are not evenly distributed among days, we randomly chose only one location per day for climatic niche analysis.

### 2.3 Fitting sigmoid curves to estimate spring and autumn migration metrics

**FIGURE 1:**
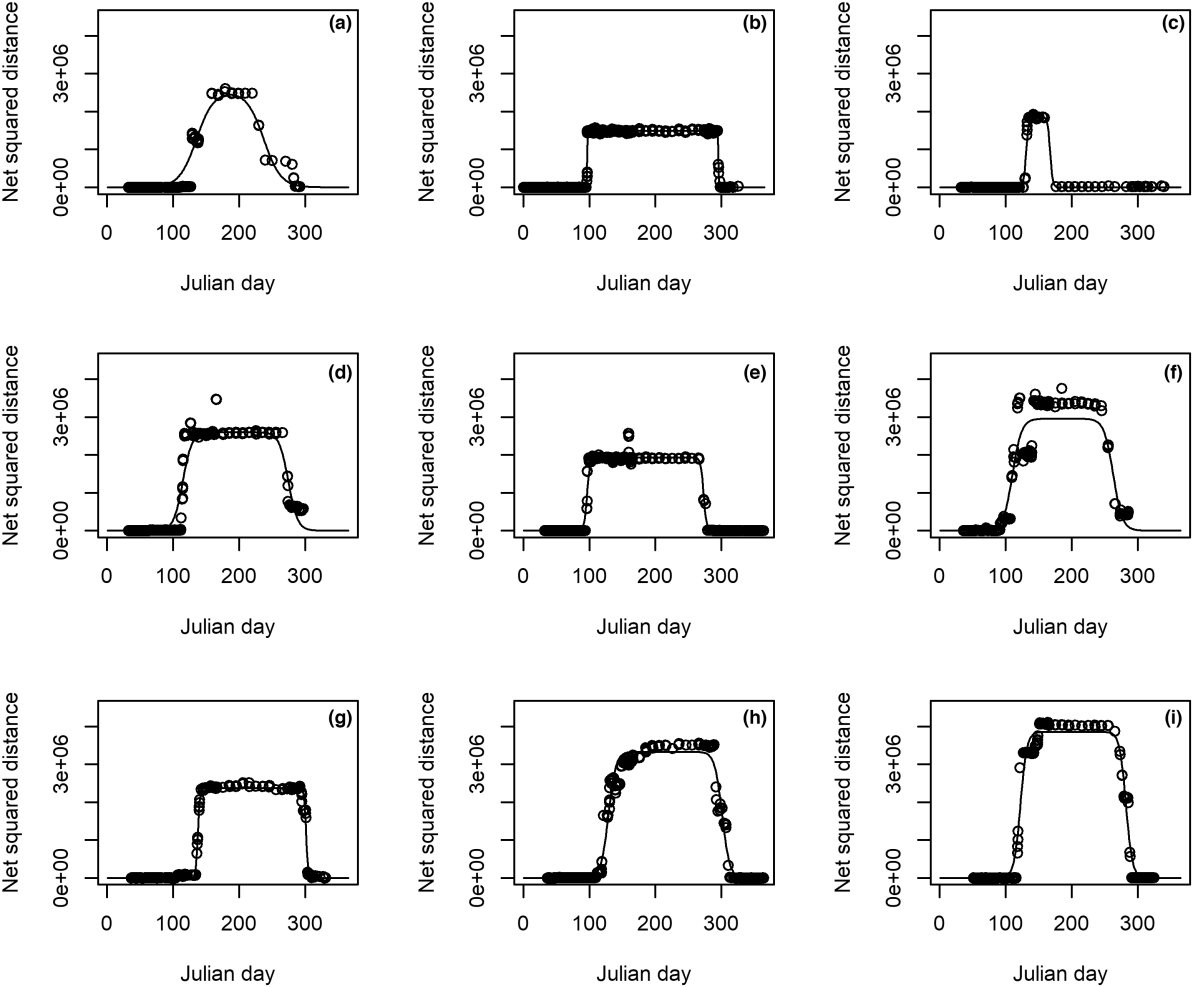
Double sigmoid curves (solid line) fitted to net squared distance time series (black solid dots) of nine double created cormorants of (a) ID 22, (b) 34, (c) 47, (d) 48, (e) 49, (f) 50, (g) 51, (h) 52, and (i) 53.

Accurate determination of migration timing and divisions of seasonal locations (i.e., on the breeding or non‐breeding grounds) are important for quantifying differences in climatic conditions that cormorants are subject to for climatic niche overlap. We reanalyzed the satellite location data on the cormorants from King et al. (2012) to estimate migration timing and movement metrics using double sigmoid models and net squared distance (NSD) methods (Figure 1; Bunnefeld et al., 2011; Soriano‐Redondo et al., 2020). Then, we examined whether the migration timing from the double logistic equation was positively related to the migration dates estimated by the NSD method. Double sigmoid curves can be used to represent annual migration trajectories of NSD. Net squared distance is the squared distance between a location and the originating location of a trajectory. Net squared distance within home ranges is stationary over time, rapidly increases after spring departure, becomes stationary again within summer home ranges after spring arrival, and rapidly decreases after autumn departure until individuals return to the non‐breeding grounds. Changes in NSD during the annual cycle can be depicted by the double sigmoid curve (Equation 1, Bunnefeld et al., 2011):

(1) $${\mathrm{NSD}}_{t}=\frac{\delta }{1+e^{\left(\frac{\theta_{s}-t}{\varphi_{s}}\right)}}+\frac{\delta }{1+e^{\left(\frac{\theta_{a}-t}{\varphi_{a}}\right)}},$$

where NSD is net squared distance on day *t*; *δ* the asymptote of NSD; $\theta_{s}$ and $\theta_{a}$ the migration time when the half of the NSD asymptote is reached in spring and autumn, respectively; $\varphi_{s}$ and $\varphi_{a}$ the time elapse between the half and ¾ of migration during spring and autumn, respectively; *t* the Julian day since the first day of a year (Bunnefeld et al., 2011). Metric 2*φ* represents the time elapse between ¼ and ¾ of migration (Bunnefeld et al., 2011). We fit double sigmoid curves to the NSD time series of nine cormorants, which had location data on both spring and autumn migration.

Twelve cormorants only had location data from non‐breeding grounds to breeding grounds because their transmitters stopped data transmission between spring arrival and autumn departure. One of the 12 birds did not have enough locations on breeding grounds for analysis. We fit the following sigmoid equation to spring migration NSD time series of 11 cormorants (Equation 2):

(2) $${\mathrm{NSD}}_{t}=\frac{\delta }{1+e^{\left(\frac{\theta -t}{\varphi }\right)}},$$

where NSD is net squared distance on day *t*; *δ* the asymptote of NSD; *θ* the migration time when the half of the NSD asymptote is reached in spring; *φ* the time elapse between the half and ¾ of migration during spring; *t* the Julian day since the first day of a year (Bunnefeld et al., 2011). Metric 2*φ* represents the time elapse between ¼ and ¾ of spring migration (Bunnefeld et al., 2011). We used R nonlinear least square function *nls* to fit the curves and estimate the unknown parameters. We used the square root of *δ* as a surrogate of migration distance and 2*φ* as a surrogate of migration duration and regressed them on migration distance and migration duration determined from the following annual movement trajectory segmentation. The model‐based metrics may compensate for the effects of coarse relocation schedules (e.g., > 1 day).

### 2.4 Determination of migrating timing and trip distance

We used the function *as.ltraj* in the R package *adehabitatLT* to calculate the NSD time series for each of 22 satellite‐tracked cormorants (Calenge, 2006). We used the R function *identify* to determine the beginning of spring NSD increase as spring departure, the beginning and ending of the NSD summer plateau as spring arrival and autumn departure, and the ending of autumn NSD decreases as autumn arrival at non‐breeding grounds (King et al., 2017; Soriano‐Redondo et al., 2020). Those positions were used to determine the dates of the corresponding migration timing with the satellite tracking timestamp.

Migration trip distance was calculated as the sum of all step segments along spring or autumn migration using the function *as.ltraj*. The difference in days between spring departure date and spring arrival date was used as spring migration duration, whereas the difference in days between autumn departure date and autumn arrival date was used as autumn migration duration. The satellite tracking used in this step did not render regular time intervals between two successive locations. We calculated hourly movement distance for winter, spring migration, and summer using movement step length between two successive locations, which had time intervals of 30–60 min. Hourly distance was calculated by dividing step length (km) by time interval in hour.

### 2.5 Estimation of climatic niche overlap between winter and summer

We obtained climatic variables from the WorldClim (version 2) database (Hijmans et al., 2005; http://www.worldclim.org), which provides a variety of monthly climatic data and wind speed averaged over the years 1970–2000 covering both Canada and the US. The spatial resolution of the WorldClim data used in this study is 30 arc‐second (i.e., 1/3600 degree) of longitude and latitude, about 1 km which was sufficient for our analysis.

We derived seasonal mean monthly minimum and maximum temperature (°C), seasonal mean monthly precipitation (mm), and seasonal mean wind speed (m/s) from November to February for the non‐breeding grounds and from June to August for the breeding grounds. The arrival and departure dates of cormorant seasonal migration vary substantially from year to year. The two periods were chosen because they correspond to the time of year when most migrating cormorants are present on the non‐breeding and breeding ranges, respectively (King et al., 2012; Scherr et al., 2010).

We estimated climatic niche overlap between winter and summer using an ordination method based on principal component analysis (PCA; Broennimann et al., 2012). The ordination approach compares seasonal niches in the environmental space represented by the first two principal components (PCs) of PCA. We used the thinned location data (>1‐day time interval between successive locations) for cormorant presence on the breeding grounds during summer and on the non‐breeding grounds during winter. Species occurrences are first mapped to a grid of 100 × 100 cells on the environmental space; then, occurrence density is estimated by kernel smoothing methods for each cell and is rescaled to the range from 0 to 1 (Broennimann et al., 2012). Climatic condition is also smoothed and rescaled to the 0–1 range for each grid cell (Broennimann et al., 2012). Therefore, the ordination approach allows for direct niche comparisons between regions or between seasons considering differences in climatic condition and geographic extent. The ordination method can also visualize ecological niche overlap between the breeding and non‐breeding seasons in the first two PCs of the environmental space. We used the R package *ecospat* to conduct the ordination analysis of cormorant seasonal niches (Di Cola et al., 2017). We used the *I* statistic and Schoener's *D* to measure niche overlap (Schoener, 1968; Warren et al., 2008). Indices *I* and *D* range from 0 to 1, with 0 indicating no niche similarity between seasonal niches and 1 indicating complete similarity.

## 3 RESULTS

Estimates of spring migration metrics *δ*, *θ*, and *φ* by the single sigmoid equation for 11 cormorants are presented in Table 1. The estimates of spring and autumn migration metrics *δ*, *θ*_s_, *θ*_a_, *φ*_s_, and *φ*_a_ and model fits are presented in Table 2 and Figure 1. Location data on autumn migration trips were sparser than those on spring migration trips (Figure 1). Among nine tracked cormorants of both spring and fall migration, two transmitters stopped data collection before autumn migration ended (Figure 1d,f) and three transmitters rendered sparse data during autumn migration (Figure 1c,e,g). Therefore, observed hourly speed and the total trip distance measured from tracking autumn migration trajectories were not included in the analysis.

**TABLE 1:** Parameters of double sigmoid curves depicting spring and autumn migration of nine Double‐crested Cormorants captured in Alabama, Arkansas, Louisiana, and Mississippi, United States from 2000 to 2001.

ID	Asymptote *δ*_s_ (km^2^)	*δ*_s_ SD	Mid‐point *θ*_s_ (day)	*θ*_s_ SD	Mid‐point *θ*_a_ (day)	*θ*_a_ SD	Time elapse *φ*_s_ (day)	*φ*_s_ SD	Time elapse *φ*_a_ (day)	*φ*_a_ SD
22	2542838.60	48650.29	135.28	0.50	237.15	1.37	6.14	0.45	13.01	1.28
34	1488701.72	791.86	96.66	0.00	295.36	0.01	0.08	0.00	0.45	0.01
47	1861991.00	2897.89	131.11	0.02	167.39	1.00	0.83	0.01	1.93	0.22
48	2598861.00	12171.25	114.57	0.04	274.45	0.43	0.41	0.07	6.96	0.41
49	1934719.44	5660.00	96.54	0.02	272.86	0.22	0.27	0.03	2.23	0.23
51	2958255.99	48409.27	110.30	0.62	262.93	1.85	2.88	0.54	6.92	1.28
52	2376075.74	8024.31	137.44	0.08	301.32	0.10	1.42	0.06	1.53	0.09
53	3329986.49	25540.34	128.87	0.40	301.01	0.47	7.24	0.36	5.64	0.40
54	3857982.16	24525.05	122.42	0.35	282.24	0.35	3.37	0.22	4.85	0.32

*Note*: Initial SD stands for standard deviation. Subscripts s and a stand for spring and autumn.

**TABLE 2:** Parameters of single sigmoid curves depicting spring migration of 12 Double‐crested Cormorants captured in Alabama, Arkansas, Louisiana, and Mississippi, United States from 2000 to 2001.

ID	Asymptote *δ* (km^2^)	*δ* SD	Mid‐point *θ* (day)	*θ* SD	Time elapse *φ* (day)	*φ* SD
5	5355946.16	55251.13	125.77	0.22	3.64	0.21
16	4721621.13	62610.51	128.40	0.59	8.43	0.52
17	2429528.95	20273.21	131.06	0.27	5.58	0.26
18	2093731.29	18330.90	130.27	0.21	3.02	0.20
20	2718863.72	24973.17	108.66	0.30	6.02	0.24
21	2433822.63	14393.77	112.55	0.16	2.98	0.13
26	2090535.46	16638.20	140.29	0.51	7.60	0.48
27	1622824.09	8397.61	129.27	0.05	0.67	0.06
28	3176429.01	22532.12	112.09	0.18	2.30	0.17
33	2658532.82	5670.81	112.02	0.05	1.28	0.04
36	1979773.66	3402.35	102.57	0.04	1.18	0.03
46	1260050.02	15834.24	114.85	0.36	2.53	0.34

*Note*: Initial SD stands for standard deviation.

Average hourly movement distance did not differ between winter (averaging 4.09 km and ranging from 0.59 km to 16.3 km, *n* = 21) and summer (averaging 3.32 km and ranging from 0.15 km to 6.8 km, *n* = 21) with their 95% confidence intervals (CIs) overlapping (Figure S1a). Observed spring migration durations averaged 23.32 days, ranging from 1.8 days to 61 days (*n* = 22), whereas the time elapsed between ¼ and ¾ of spring migration (2*φ*_*s*_) averaged 7.39 days, ranging from 0.45 day to 27.12 days (*n* = 21). Observed spring migration duration was positively related to model‐based migration spring duration (*β* = 2.67, *R* ^2^ = .73, Figure S1b). The observed total trip distance of spring migration (averaging 1161.08 km and ranging from 996.24 km to 2391.19 km, *n* = 20) was positively related to mean asymptote distance of spring migration (averaging 1599.84 km and ranging from 1129.52 km to 2314.29 km, *n* = 20, *β* = 0.37, *R* ^2^ = .35, Figure S1c). Average autumn migration duration was 22.71 days (95% CI: 4.13–41.3 days, *n* = 7) and was comparable to that of spring migration (mean = 23.32 days; 95% CI: 16.05–30.58, *n* = 22). Time elapse between ¼ and ¾ of autumn migration was positively related to observed autumn migration duration (slope *β* = 4.6, SE = 0.35, *R* ^2^ = .97). Average total trip speed was not related to hourly speed during spring migration (Figure S1d, *n* = 19).

**FIGURE 2:**
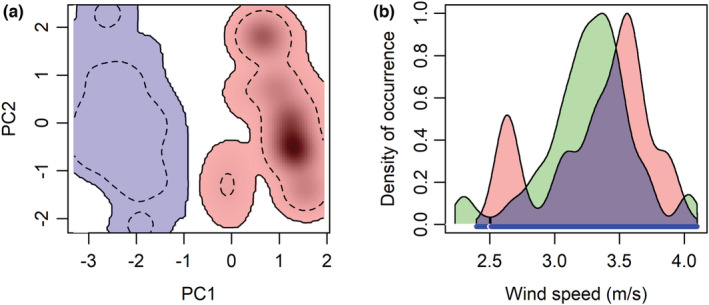
(a) Seasonal climatic niche switching of Double‐crested Cormorants with the polygon of blue color representing wintering‐ground climate niche and with the polygon of red color representing breeding‐ground climatic niche. The inner black dashed contour lines represent 50% (most common) of climatic conditions. Polygons represent climatic niche in the climate sub‐space depicted by the two principal components (PC1, PC2). PC1 explains 71.43% of the total variability and represents high precipitation. PC2 explains 25.38% of the total variability and represents low wind speed. (b) Seasonal similarity of wind speed between summer and winter with the polygon area of blue color representing similarity of wind conditions between the breeding (green polygon) and non‐breeding season (red).

Winter climatic niche was separated from summer climatic niche (Figure 2a), with indices *I* and *D* being zero. Except for wind speed (Figure 2b), the observation ranges of precipitation, minimum temperature, and maximum temperatures overlapped little between the breeding and non‐breeding grounds (Figure S2). Cormorants appeared to be subject to similar wind conditions for their winter and summer movements (Figure 2b; winter: mean speed = 3.33 m/s, standard deviation [SD] = 0.4; summer: mean = 3.26 m/s, SD = 0.34).

## 4 DISCUSSION

Avian migrants face unique challenges by the increasing energetic costs of flight and the demand of the optimal use of wind energy for migratory and local movements (Butler & Bishop, 2000; Hedenström, 1993; Safi et al., 2013). Satellite or GPS tracking technologies enable animal ecologists to investigate seasonal variation in migration and climatic niche of the same populations (Harel, Horvitz, & Nathan, 2016). Our findings support the hypothesis that cormorants are climatic niche switchers between their breeding grounds in the northern temperate region and non‐breeding grounds in the subtropical region. On the contrary, cormorants used similar wind conditions for non‐migratory movements on the breeding and non‐breeding grounds.

Migratory birds may either track similar niches or switch to different niches to cope with physiological challenges and resource availability between summer breeding grounds and winter non‐breeding grounds. Previous studies have found evidence for avian climatic niche trackers (Gómez et al., 2016; Joseph & Stockwell, 2000; Nakazawa et al., 2004) and climatic niche switchers (Laube et al., 2015; Marini et al., 2013). Climatic conditions were colder in the summer habitat occupied by cormorants than in the winter habitat (Figure S2). Although avian migration is affected by several other factors such as competition, predation, and genetics, climate plays a unique role in driving the autumn migration of fish‐eating birds nesting in the northern temperate regions. Much colder autumn temperatures at the northern temperate breeding grounds may drive avian migrants to migrate southward to subtropical or tropical regions to enhance winter survival (Gómez et al., 2016; Somveille et al., 2015). Cormorants forage in rivers and shallow lakes; thus, cold winter temperatures may freeze the surface water of shallow water bodies at the nesting grounds, preventing cormorants from acquiring sufficient food and forcing them to migrate southward in late autumn. The data on the migration of the cormorants used in this study were collected 20 years ago. Ambient air temperatures have increased with a projection of more precipitation in North America due to current climate changes (IPCC, 2021). It is uncertain whether climate changes have modified the migration patterns and climatic niche of the cormorants. Future studies are needed to investigate the effects of climate changes on the probability and distance of spring migration of the cormorants from the southeastern US.

Our estimates of total travel distance of spring migration were greater than those in King et al. (2012). A possible reason for the difference is that King et al. (2012) used the first location of spring and autumn migration as the departure days and recognized that their estimates might be a few days later than the actual initiation days. Second, we used the curve of NSD during the annual migration to determine the departure dates with the wintering and summer ranges being delineated for each bird. Our study demonstrated that the combination of model‐based migration metrics and metrics determined from track segmentation may allow us to make inference of the migration timing if satellite tracking data are sparse. The number of days en route and time elapsed between the ¼ and ¾ migration were closely related during both spring and autumn migration of cormorants. Additionally, the total migration distance summing over all step segments along a migration route was also positively related to the asymptote NSD from double sigmoid curves. Furthermore, asymptote distances estimated by double sigmoid curves fall in the range (1000–2000 km) of the maximum travel distances estimated using band recovery data (Dolbeer, 1991). The consistency between the two approaches suggests that our determination of the summer and winter habitats used by cormorants and the climatic conditions of the habitats for climate niche analysis are reliable. The 22 tracked cormorants of this study were captured in Alabama, Arkansas, Louisiana, and Mississippi and migrated along the Central, Mississippi, and Atlanta Flyways (Figure 1 of King et al., 2012). It is not known whether the tracked cormorants exhibited fidelity to the same flyways each year. Additionally, it is uncertain how differences in the breeding areas among the 22 cormorants affect the estimation of seasonal climate niche variation.

Wind plays a critical role in determining avian flying performance (Pennycuick, 2008; Safi et al., 2013). Despite non‐overlapping summer and winter climatic niches (Figure 2a), cormorants appeared to be subjected to similar wind speed between winter and summer (Figure 2b). The similar wind conditions between the summer and winter habitats occupied by cormorants were consistent with similar hourly flying speed between winter and summer. Although we reduced location error from the Argos tracking data using state space models, coarse temporal and spatial resolution of our data prevented us from investigating the effects of tailwind speed on the flying speed of cormorants like Illan et al. (2017) and Safi et al. (2013) or estimating selection for a certain range of wind speeds using a resource selection function like Nourani et al. (2018). Future studies are needed to investigate relationships between wind conditions and movement of cormorants using fine resolution relocation data. Furthermore, the age composition of our tracked birds was heavily skewed to immature birds (≤3 years old) with only six adult birds being tracked (King et al., 2012). About 6%–24% of second‐year cormorants were found breeding at the breeding grounds (Chastant et al., 2014). The sexes of our tracked birds were not determined (King et al., 2012), so we were not able to evaluate the differences in the migration and climate niche between the sexes. Future studies are needed to address the effects of ages and sexes on the migration ecology and seasonal climate niches of cormorants.

## AUTHOR CONTRIBUTIONS

**D. Tommy King:** Conceptualization (equal); data curation (equal); investigation (equal); writing – original draft (equal). **Guiming Wang:** Conceptualization (equal); formal analysis (equal); methodology (equal); visualization (equal); writing – original draft (equal); writing – review and editing (equal). **Fred L. Cunningham:** Conceptualization (equal); investigation (equal); project administration (equal); supervision (equal); writing – original draft (equal); writing – review and editing (equal).

## CONFLICT OF INTEREST

The authors have no conflict of interest related to this work.

## Supporting information


Figures S1–S2

## Data Availability

Data used in this study are available at the Dryad Digital Repository. https://doi.org/10.5061/dryad.z8w9ghxfr.
