# Supplementary material for: Seasonal climatic niche and migration movements of Double‐crested Cormorants
Source: Ecol Evol. 2022 Aug 23;12(8):e9153. doi: 10.1002/ece3.9153 (PMC9396706; doi:10.1002/ece3.9153)

Seasonal climatic niche and migration movements of Double-crested cormorants

Tommy King^1^, Guiming Wang*,^2^, and Fred L. Cunningham^1^

^1^U. S. Department of Agriculture, Wildlife Services, National Wildlife Research Center, Mississippi State, Mississippi USA; E-mail: [dtking1018@gmail.com](mailto:dtking1018@gmail.com); [Fred.L.Cunningham@usda.gov](mailto:Fred.L.Cunningham@usda.gov). ORCID ID: 000-0002-6035-3313

^2^Department of Wildlife, Fisheries and Aquaculture, Mississippi State University, Mississippi State, Mississippi, USA; Email: [guiming.wang@msstate.edu](mailto:guiming.wang@msstate.edu). ORCID ID: 0000-0001-5002-0120

**Supplemental materials**

This supplemental material includes two figures Figs S1-S2, which were cited in the text, on the following two pages.

Figure S1. (a) Winter, spring migration, and summer hourly movement speed, (b) relationship between spring migration duration (days, y axis) and time elapse between ¼ and ¾ of spring migration (x axis), (c) relationship between asymptotic spring migration distance (y axis) and the total spring satellite track distance (x axis), and (d) relationship between total migration speed (y axis) and hourly speed (x axis) of Double-crested cormorants.


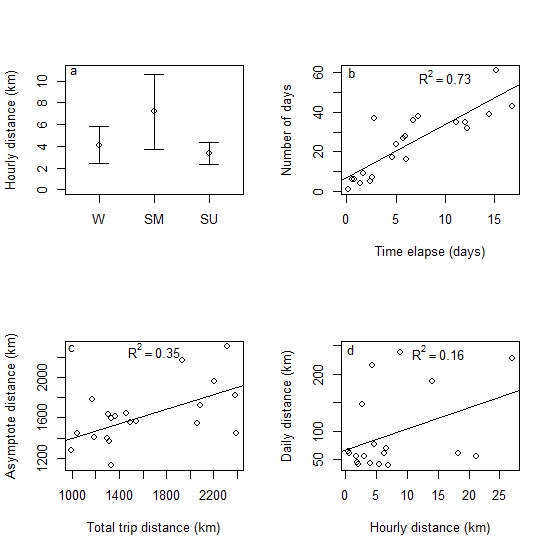


Figure S2 Frequency distribution histograms of mean monthly precipitation, minimum temperature, maximum temperature, and wind speed on winter (November to February) and summer (June to August) habitat occupied by Double-crested cormorants.


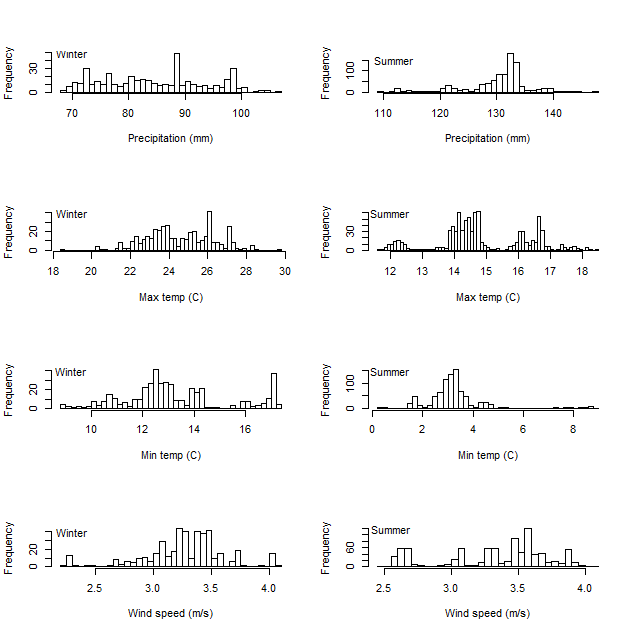

Supplement: Supplementary file 1 — Figures S1–S2 [file ECE3-12-e9153-s001.docx]
